# Women’s Breast Cancer Knowledge and Health Communication in the United Arab Emirates

**DOI:** 10.3390/healthcare8040495

**Published:** 2020-11-18

**Authors:** Dania Abu Awwad, Syeda Zakia Hossain, Martin Mackey, Patrick Brennan, Shukri Adam

**Affiliations:** 1 Faculty of Medicine and Health, The University of Sydney, Camperdown, NSW 2006, Australia; dabu4509@uni.sydney.edu.au (D.A.); martin.mackey@sydney.edu.au (M.M.); patrick.brennan@sydney.edu.au (P.B.); 2 College of Nursing, Ras Al Khaimah Medical & Health Sciences University, Al Qusaidat, UAE; shukri@rakmhsu.ac.ae

**Keywords:** breast cancer, health promotion, screening, health education, women’s health services, United Arab Emirates

## Abstract

In the United Arab Emirates (UAE), women’s participation in breast cancer screening is low, and women are commonly diagnosed in advanced stages. This study investigated women’s attitudes towards breast cancer screening, their use of health services in the UAE emirate of Ras Al Khaimah, and their preferred medium for breast cancer information. In this qualitative study, six focus groups were conducted with Emirati (*n* = 28) and non-Emirati (*n* = 26) women as Ras Al Khaimah is a highly multi-cultural region. Women were separated into different age groups (25–34, 30–44, 44+) so as to obtain perspectives of young (*n* = 16), middle (*n* = 19), and older women (*n* = 19). The focus group transcripts were analysed using thematic analysis. Women recognised that any breast change should be checked by a doctor, and that women with symptoms or those at higher risk may need to have breast screening earlier than the recommended starting age. However, participants wanted more information from doctors or other health personnel. Women had observed breast cancer information and campaigns advertisements in multiple media but recommended greater use of social media and WhatsApp to disseminate information. Overall, women had positive attitudes towards breast cancer screening but wanted more breast cancer awareness campaigns year-round and better access to screening.

## 1. Introduction

Breast cancer is the most common cancer in women in the Gulf countries and the United Arab Emirates (UAE) and the leading cause of cancer death [1,2]. Women are commonly diagnosed with breast cancer in advanced stages, with studies finding that two-thirds of breast cancer cases were advanced at diagnosis in Saudi Arabia [3,4]. In Oman, just under 50% of cases were diagnosed at stage III [5], while in the Gulf Cooperation Countries, almost 60% of cases were diagnosed at advanced stages with regional or distant metastasis on diagnosis [6]. In a study in the northern emirates of the UAE, while the majority of cases were diagnosed with stage II, 16% were diagnosed at stage III and a third of all cases had lymph node involvement [7].

Breast cancer has a high survival rate of 98% if detected early [8]. However, when women delay seeking medical attention after noticing changes in their breast, they are more prone to have a late breast cancer diagnosis with a larger tumour in an advanced stage, and lower survival rates [9]. A study in Saudi Arabia found that women waited approximately four months between finding breast changes and seeing a doctor [10]. More than half of these women were diagnosed with a tumour in advanced stages [10]. Limited knowledge about the signs and symptoms, screening and diagnostic services, and treatment of breast cancer has led to delays in medical attention for breast cancer in the Middle East [9]. A common issue in the region as well is the lack of recommendations and health education from healthcare professionals for preventative care and breast cancer screening [11,12]. Early studies in the UAE have found that less than 15% of women aged 40–65 years old practiced breast self-examinations (BSE), clinical breast examinations (CBE), or had mammograms [13]. Recent studies continue to show low levels of breast cancer knowledge and low breast screening participation rates in the Gulf countries [14,15,16], and in Ras Al Khaimah (RAK), a northern emirate of the UAE [17].

Understanding women’s breast cancer knowledge in a region can help discern what needs to be addressed, and reducing structural barriers has favourable results on women’s participation in screening [18]. This would then highlight methods to enable women to participate in screening, increase participation rates, and increase early detection rates of breast cancer. These previous studies have used quantitative methods to gain an overview of women’s attitudes in the UAE and neighbouring countries. Within this region, there are limited studies using a qualitative approach to gain a deeper understanding of women’s attitudes and barriers towards breast cancer screening [9,19]. Studies exploring women’s knowledge, screening behaviours, and barriers have subsequently recommended qualitative approaches [17,20]. Hence, the aim of this paper is to find out the knowledge and attitudes of women towards breast cancer and screening in RAK in greater depth. With that, the aim is to gain an understanding of women’s use of health services, how they receive breast cancer information, and what are the best communication strategies to encourage women to participate in breast cancer screening.

## 2. Materials and Methods

A qualitative study was conducted with women living in RAK in line with the Declaration of Helsinki. Ethics approval was obtained from the University of Sydney’s Human Research Ethics Committee (2014/884), and participants signed a written consent form in either English or Arabic. Six focus group discussions (FGDs) with semi-structured questions were held with Emirati and non-Emirati women, and separated into different age-groups (25–34, 30–44, and 44+ years old). Convenience sampling methods were used for recruitment, and the study was also advertised online and through posters. Data were collected on participants’ socio-demographic background using a short survey. The FGDs covered topics on breast cancer knowledge questions, beliefs and attitudes regarding breast cancer and screening, women’s health services, and preferred sources of information and promotion. The FGDs were audio recorded and subsequently transcribed verbatim, coded using NVivo, and thematic analysis was undertaken. An Arabic transcriber and translator transcribed the Arabic audio recordings, and these were checked by the bilingual researcher.

The Chi-square test was used for statistical comparison between the Emirati and non-Emirati participants, and the significance level was set at a *p*-value of 0.05. The Statistical Package for the Social Sciences was used to conduct the statistical analysis.

## 3. Results

Fifty-four women—28 Emirati and 26 non-Emirati—participated in the FGDs. Three FGDs included Emirati women and 3 included non-Emirati women, with both groups of women broken into three different age-groups (25–34, 30–44, and 44+ years old). Two groups contained one participant that did not fit into those age-groups but were not excluded and were permitted to participate. Four FGDs were conducted in English. An FGD was in Arabic for the Emirati women aged 44+ years old, and in both English and Arabic simultaneously for the Emirati women aged 25–34 years old. There were 8–11 women allocated to each group. Each FGDs required between 30 and 60 min to conduct.

Table 1 presents the demographic characteristics of the participants. Participants were 25–65 years old, with an average age of 39 years old. In total, 88% of women in the 25–34 age-group were students. Four out of the seven (57%) Emirati women with a high school level of education were university students. The Chi-square test was used to compare the differences between the demographic factors of the Emirati and non-Emirati participants. The variables were independent (*p* > 0.05) for all demographic factors except for family history of breast cancer (*p* = 0.024) where there were more non-Emirati women with a family history of breast cancer than Emirati women.

### 3.1. Breast Cancer Knowledge

Each group listed a range of breast cancer symptoms, with an emphasis on lumps, but summarised breast cancer symptoms as any changes or abnormalities noticed in the breast. Each group identified BSE, CBE, and mammography as the three most common types of breast screening, and were aware of the benefits of screening such as early detection, reduced mortality, and simpler treatment. Other common diagnosis methods identified included biopsies, ultrasound, and blood tests. Less commonly known screening methods were CT scans, tumour markers, and thermography. In general, older women provided more detailed information about the examinations and mammography than the groups containing younger women.

Table 2 presents participants’ responses about BSE, CBE, and mammography. Some Emirati participants from the 30–45 and 44+ age-groups described the BSE steps, and all groups knew that BSEs should be performed monthly after the menstrual cycle. However, a few participants stated that research has demonstrated that it was important not to depend on BSE alone as it is associated with high false positive rates.

Researcher: “*Have you heard about false positive or overdiagnosis?*”

002: “*That’s why I actually I said in the beginning that it is not recommended to be doing self-breast examination because of that now. The latest studies that I attended, lectures too, they said breast self-examination is not recommended anymore. But everyone can decide*.”

(30–44-year-old non-Emirati)

The participants recommended getting women to practice BSE and CBE while young so that they become comfortable with their body and getting examined by doctors (see Table 2). Most women knew that mammography is for women aged over 40, but participants explained that women with higher risk would have mammograms earlier and more frequently, and symptomatic women would have mammograms regardless of age.

Many women in each group had practiced BSE, but not all of them practiced it monthly. Few women did not practice BSE at all. Younger groups were more likely to list reasons for having CBEs or mammograms such as having symptoms, fear, or feeling encouraged after seeing an awareness program, whilst the older age-groups had CBEs more regularly or during their regular health check-ups. Most women in the 44+ age-group had mammograms. Those who did not stated that they encourage other women to participate, but personally neglect themselves. Participants less than 40 years old explained that they did not have mammograms because they were not in the target age-group, were asymptomatic, and not at increased risk but would have mammograms if advised. Women encouraged other reluctant participants by reminding them of the benefits of screening:

006: “*This is our nature. We may not personally go but we encourage others, and say you must go and see*.”

008: “*Like, it hurts me but I am checking to see if I have a disease which is better than having the disease and letting the disease grow, and then I have a mastectomy, removing my first and second breast, and for it to spread in my body. What would I have gained thereafter? I would not have gained anything. … It’s true that the mammogram hurts but it is an examination. I don’t have anything, thank God, this is a good outcome to come out with beautiful news*.”

(44+-year-old Emirati women)

### 3.2. Beliefs and Attitudes Regarding Breast Cancer and Screening

Participants recognised the ambiguity or indefinite nature of factors that can cause or increase the risk of cancer, and were familiar with hereditary and lifestyle factors. Women from all groups clarified that they do not believe that there are religious or cultural beliefs that cause breast cancer. Some non-Emirati women mentioned that people in their home country might have different attitudes towards health. For example, a non-Emirati woman stated that people maintain unhealthy behaviours because they believe that they are going to get cancer because they live in a high-risk area. Another non-Emirati woman stated that people only go to the doctor with symptoms, and not for preventative services:

006: “*When I lived there, they will believe in that all of us will get some kind of cancer, and of course all ladies there are really very well aware about it, and they’re expecting it. Like anyway it will happen so I can smoke for example. I will not quit, because anyway it will happen because we got Chernobyl catastrophe in 1986*.”

(30–44-year-old non-Emirati)

The older Emirati women did mention superstitions such as the “evil eye” and envy, and that it is important to be mindful of God. They were also curious to know whether there was any scientific backing for healing properties of some fruits or trees. The participants clarified that stories or substances that people “swear by” do not prevent them from seeing the doctor or having treatment in hospitals. However, they recounted stories of people they knew who sought earlier treatment in Western countries, told that there was no hope, but were later “cured” after using traditional medicines. As a result, participants would use traditional medicines out of desperation.

Researcher: “*From the things that you’ve heard, does it prevent you from going to the doctors, having treatment in hospitals, or do you try both*?”

006: “*No, no, no*”

004: “*All this has nothing to do with us*”

009: “*To be honest, we are not convinced by this stuff*”

(44+-year-old Emirati women)

### 3.3. Women’s Health Services

The 25–34-year-old Emirati women were more familiar with public and private women’s health services than non-Emirati women of the same age-group, but mothers or older age-groups used those services more. Emirati women preferred public services over private services, mentioning that they have free access and greater trust in the public system. However, there were long waiting times to get an appointment for specialists which were prioritised according to severity, and as a result, women might go to another emirate or pay to use private services. Private health services were predominantly used by non-Emirati women. Women stated they would go to other emirates if services were not available in RAK, for more affordable clinics, or if specific doctors or places had stronger reputations:

008: “*Not available in Ras Al Khaimah, we’ll go to another city. Like infertility, not available here, we’ll go to another city like Dubai*.”

(30–44-year-old Emirati)

Screening is not covered by all insurance coverages, and mammograms were found to be expensive, but they can receive free or subsidised screening through hospitals, clinics, or the national breast screening awareness program “Pink Caravan”, for example,

004: “*Generally, I wait because my insurance does not cover screening, as I said. I do wait for the month of October. Because so many companies and clinics are taking part in that now, and they have offers that I can live with. So I tend to do my screening then*.”

002: “*And if it’s annually, you’re still covering your annual check-up*.”

(44+-year-old non-Emirati)

### 3.4. Sources of Information and Promotion

The internet and social media were the most common forms of information identified regardless of age-group. Pamphlets were the most common paper-based format for breast cancer information distributed in hospitals, primary healthcare centres (PHCCs), and malls. Doctors and gynaecologists were the most commonly referred individuals to talk to the participants about breast cancer and screening. The Ministry of Health and Prevention (MOHAP) has also been identified as a common source of information and advertisements for breast cancer events. See Table 3 for sources of information and advertisements.

All groups are aware of various breast cancer awareness campaigns in RAK (see Table 4). There are occasional events throughout the year, but there was a heavy focus on the October month during which awareness campaigns and screening services are held everywhere. Pink Caravan was the most identified initiative, and MOHAP was mentioned several times and praised for its involvement in awareness campaigns.

Table 5 lists the preferred media or activities recommended by the participants. Social media and WhatsApp were heavily encouraged as a form of communication method to raise awareness and share upcoming breast cancer events and screening. It is something that was used by everyone, regardless of age-group.

## 4. Discussion

Authors should discuss the results and how they can be interpreted in light of previous studies and of the working hypotheses. The findings and their implications should be discussed in the broadest context possible. Future research directions may also be highlighted.

The FGDs with women highlighted their attitudes towards participation in breast screening and how best to raise awareness on breast cancer among women in RAK. The participants use of women’s health and breast screening services varied between age-groups, with greater use among middle and older groups which also gave them greater familiarity with the types of services available in RAK. Health professionals within health centres and social media were deemed important and effective means of communication in improving breast cancer awareness and breast screening rates by the participants.

### 4.1. Breast Cancer Knowledge

Women were familiar with a variety of breast cancer symptoms and diagnosis methods and recognised that any change in the breast should be discussed with a doctor. However, previous studies in neighbouring countries have found that women were not always familiar with BSE and CBE [21,22]. One study in Saudi Arabia found that the majority of participants believed that women over 20 years old should be participating in BSE monthly and be educated about it although two-thirds of women have not heard about BSE [21]. Another study found that less than half of the participants had heard of BSE and less than a third of CBE [22]. However, in this study, some Emirati participants in the 25–34 age-group stated that they had not heard the term ”clinical breast examination” but suggested that it must mean a doctor or medical professionals checking the breast. They were also able to state places or screening programs that conduct CBEs. This suggests that people might be familiar with screening examinations but not familiar with their medical terms, which could also be due to language limitations when communicating in English with Emirati participants.

Participants varied in knowledge concerning the starting age and frequency of BSE, CBE, and mammography, but their responses were similar to the MOHAP breast screening guidelines [23]. The variations could be due to uncertainty or non-Emirati women referencing the screening recommendations from their home countries.

Studies in the region and UAE have reported that between 20 and 50% of women practiced BSE [24,25,26,27,28]. Another study found that approximately 75% practiced BSE but less than a quarter did so correctly and regularly [29]. This finding is more reflective of the responses from the participants in this study, with many saying they practiced BSE, but some admitted that they are not regular. It was not uncommon for women to purposefully perform examinations if they have symptoms or pain, or to check their whole breast after noticing a change while bathing.

Studies have similarly found low participation rates for CBE and mammography. Less than 15% of the target age-group had CBEs [13] or mammograms [16] in the UAE. Within this study, most of the women in the 44+ age-groups had had CBEs and mammograms. Younger women explained that they did not have mammograms because they were not part of the target age-group and had no symptoms, but those who did justified their participation due to having symptoms. Women who had never had a mammogram complained more about the associated pain than women who did participate. In response, the hesitant women were informed by other FGD participants that they have to try it out themselves when it becomes time to start having mammograms, or told them that they needed to have a mammogram now if those women were in the 44+ age-group. This encouragement between women shows a positive attitude towards breast screening across the different age-groups, with some women acknowledging the pain associated with mammograms but believing that it is essential for the early detection of breast cancer. This finding is similar to another study in the UAE among Emirati and non-Emirati women, where women who had regular screenings had more positive attitudes towards health services and believed in the importance of early detection [30].

### 4.2. Beliefs and Attitudes Regarding Breast Cancer and Screening

Fate is commonly cited as a barrier to breast screening in previous research studies [9,31], but it cannot be simultaneously connected with lack of action for one’s health unless it was clarified as such. Other authors have explained that there are variations on how women interpret beliefs of fate and destiny, which may contradict the associated religious meaning, and it may be either an enabling factor or barrier towards participating in preventative screening activities [32,33]. Women have simultaneously expressed their religious belief in fate and God whilst also recognising the role of their religion in encouraging proactive behaviour and responsibility over their health [32]. When participants in this study raised religion or cultural remedies in discussions, they were followed up with whether those practices inhibit them from seeing the doctor. They clarified that it did not but nor would they be dismissive of traditional medicines. A study among Qatari women attending PHCCs found that more than a third of these women used complementary and alternative medicines (CAM) as well [34]. In Ajman in the UAE, approximately 50% of female participants in the study used CAM and were also patients within a hospital health care centre [35]. These findings are suggestive that using cultural or natural remedies does not mean people will not seek medical attention when needed. It is also important for healthcare providers to be aware of the benefits and potential harms of CAMs and to be able to provide culturally competent care [35]. Similarly, some of the Emirati participants in this study would regularly thank and praise God or make supplications in their remarks, and in reference to the need to be optimistic, maintain a high spirit, and trust in God if diagnosed with a disease. Previous studies highlighted the importance of religious faiths in helping women with breast cancer to cope and find solace [31,36,37].

### 4.3. Women’s Health Services

Emirati women stated that they preferred public services partly because they have greater trust in the system, and also stated that they would go to other emirates if a doctor or facility has a good reputation. A study in Abu Dhabi found that women regularly having breast screenings were comfortable with the screening services available, while those who were not regularly screened mistrusted the healthcare services [30]. While it is not clear as to what came first, mistrust or not using the services, this finding suggests that it is important for women to trust the health services available in order to use them, and they were willing to travel long distances for them. For non-Emirati participants, there was a frequent comparison between the costs of services available to them in RAK and the associated costs with their home country. These women were more reliant on insurance cover. Ninety percent of the total UAE population have some form of health insurance coverage [38], but most insurances do not provide coverage for mammographic screening [39,40]. Participants in the middle and older age-groups in this study were more knowledgeable than the younger age-groups about breast screening and women’s health services available in RAK, and used them more frequently. This finding was consistent with another study in the UAE, where younger participants were not aware of where breast screening services were available, whilst older women had greater awareness [30], and this could be a result of older people’s greater use of health services.

### 4.4. Sources of Information and Promotion

The women noted that they were aware of a variety of media for promotion of breast cancer campaigns or information, and different institutions were involved in raising breast screening awareness such as hospitals, clinics, and schools. Common sources of breast cancer information identified in previous studies included the television and print media [27,41], family and friends [27,42], the radio [43], internet [44], schools [14], and public campaigns [14,44,45].

The participants recommended using social media and WhatsApp as the best media to share information on breast cancer and breast screening campaigns as it is widely used by everyone, even among the older generations. Mass media were not used by the participants themselves but encouraged because more media should attract more women. Women were keen on receiving information from doctors or at least other health personnel, and to be given the chance to ask questions. The participants showed a positive attitude towards receiving health information but needed an encouraging environment as well. A study in Turkey recommended the use of educational programs to improve screening participation and to motivate and encourage screening behaviours particularly in PHCCs [20]. Findings from previous studies have also found that few women were receiving information on women’s health from doctors; however, most women wanted information on breast cancer and screening from them [21,29,46]. More information and discussions with doctors can help reduce people’s fear around cancer [46].

### 4.5. Strengths and Limitations

The participants in this study had a good level of breast cancer knowledge which differs with previous studies from the UAE, and may be a result of selection bias. Though participants were recruited from various locations in RAK, the study sample may not be representative of women in RAK, particularly women in more rural areas. However, data saturation was achieved within the sample size, and information gathered in the first focus groups was presented to the succeeding groups to ensure validity in the data obtained and provided a range of perspectives and rationale for differences [47,48].

A strength of this study was that both Emirati and non-Emirati perspectives were included to better represent the communities in RAK and the UAE as a whole because of its diverse population. RAK is a highly multi-cultural emirate, and 80% of the UAE population is non-Emirati [38]; hence, it is important that future studies in the UAE continue to include Emirati and non-Emirati women, and this applies to the neighbouring Gulf countries to ensure fair representation of their sample population. For example, non-Emirati women had a greater concern regarding the cost of services and dependence on health insurance coverage, whilst also having lower breast screening participation compared with Emirati women [38] which, therefore, should be considered when holding breast screening campaigns for them.

Another key novelty of this study was including insights of women from different age groups and their respective concerns because women should be participating in breast examinations from 20 to 69 years old. This age range helped highlight some similarities between age groups, such as concerns over the cost of services, but also some differences such as preferred sources of information—pamphlets for older groups and internet for younger women.

The main limitation of this study was the language barrier with the Emirati groups. The FGDs for the older women (44+ age-group) was conducted completely in Arabic. In contrast, the FGDs for the younger Emirati groups were conducted in English because the participants stated that they could all speak English. In the 25–34-year-old age-group, participants would occasionally switch to speaking Arabic and needed to have the questions repeated and translated into Arabic. During the analysis, care was taken to distinguish between moments when participants did not understand the questions in English or did not know the correct response—for example, in relation to breast cancer knowledge questions. Accuracy of the thematic analysis was maintained by use of bilingual researchers, and Arabic phrases were retained in the text if their meaning would be lost upon translation.

### 4.6. Implications for Practice and/or Policy

This study has reinforced the importance of communication between patients and health professionals and doctors for patient education and encouraging participation in breast cancer screening. The findings of this study have practical implications as it will assist professionals in improving the patient-professionals relationship, including professional’s practices and effective patient care. This, in turn, will address barriers to participation, ensure women have adequate breast cancer awareness, and subsequently participate regularly in self-examinations, clinical examinations, and mammography screening.

Breast cancer screening campaigns and information need to be suitable for diverse groups of people and appeal to their target population to have a better impact. The use of social media and various methods of communication has increased potential for spreading health information and sharing campaigning events. The reputation for healthcare facilities is perceived as important, and trust needs to be established and maintained between patients, healthcare professionals, and healthcare organisations.

The role and impact of healthcare professionals and organisations in encouraging and educating women concerning the importance of breast cancer screening warrant further research.

## 5. Conclusions

This study provides new knowledge around women’s attitudes towards breast cancer screening and how to communicate with and encourage women in RAK to participate in screening. The qualitative method used in this study allowed researchers to explore in-depth the understanding and experience of women in RAK. It also allowed women to make proactive suggestions for improved awareness of breast cancer and access to screening that are relevant to them and other women in their communities in RAK. Women who had mammograms and those who did not each had positive attitudes and encouraged participation in breast cancer screening. There were several barriers to mammographic screening associated with the availability of services in RAK and insurance coverage for access to health services. The FGDs revealed the differences between how breast cancer information was received and how women preferred to be informed, and the suggestions made by women could be implemented to have better reach over women in RAK. Future studies should also focus on ensuring women in more rural communities have access to breast cancer information and screening services.

## Figures and Tables

**Table 1:** Demographics.

Variables	Sub-Groups	Emirati		Non-Emirati		Total		X^2^	df	*p*-Value
		No.	% ^a^	No.	% ^a^	No.	% ^a^			
Age Group	25–34	8	29	8	31	16	30			
	30–44	11	39	8	31	19	35			
	44+	9	32	10	38	19	35			
Level of Education	Primary School	2	7	0	0	2	4	5.534	4	0.237
	High School	7	25	3	12	10	19			
	University	16	57	16	64	32	60			
	Postgraduate	3	11	6	24	9	17			
Employment Status	Student	10	36	6	24	16	30	7.102	6	0.311
	Employed	11	39	13	52	24	45			
	Unemployed	4	14	4	16	8	15			
	Retired	0	0	2	8	2	4			
	Student+Employed	2	7	0	0	2	4			
	Other	1	4	0	0	1	2			
Marital Status	Single	9	32	11	44	20	38	1.991	3	0.574
	Married	17	61	12	48	29	55			
	Divorced	2	7	2	8	4	8			
Children	Yes	16	67	14	67	30	67	0.237	2	0.888
	No	8	33	7	33	15	33			
Nationality	Emirati	28	100	0	0	28	53			
	Arab			2	8	2	4			
	Asian			8	32	8	15			
	African			3	12	3	6			
	European			9	36	9	17			
	North American			1	4	1	2			
	Other Non-Emirati			2	8	2	4			
Residence	Urban	24	86	18	75	42	81	3.187	2	0.203
	Rural	4	14	6	25	10	19			
Menstrual Status	Premenopausal	23	92	16	67	39	80		2	
	Menopausal/Postmenopausal	2	8	8	33	10	20	4.989		0.083
Family History of Breast Cancer	Yes	1	5	7	29	8	18	7.457	2	0.024 *
	No	20	95	17	71	37	82			
	Total	28	52	26	48	54	100			

^a^ Percentage of total women who answered the question. * Statistically significant difference, *p* < 0.05.

**Table 2:** Breast cancer diagnosis participant responses.

Breast Self-Examinations	Clinical Breast Examinations	Mammography
Q. What do you know about Breast Self-Examinations?	Q. When is it recommended for women to have clinical breast examinations?	Q. When should women have mammograms?
Responses:	Responses:	Responses:
008: “*People have to after their period. It would be approximately less than four days or three days, to feel the body if there are lumps or something. They raise their hands, their armpits. If they felt something they have to go straight away to get examined to check if it is like lumps. Or, like, if it was before the period then of course not, it is lumps from the period. But after the period, if they felt lumps it could be symptoms of cancer so they have to get it examined.*” (44+-year-old Emirati) 006: “*Regular self-examination is important. Each lady should know how her breast feels like, and when you are doing self-examination at least once per month, she can notice changes*” (30–44-year-old Non-Emirati) 006: “*The self one every month. After the period*.” 007: “*If there is no symptoms they have also to do this. After period.*” (25–34-year-old Emirati)	004: “*And have a clinical examination done to get them used to it because girls, many girls or women are so shy to have this done. So I think if you train them young, and you, you know, put this in their head, this is essential then maybe they are more likely to go for it*.” … 002: “*Because it becomes part of their life rather than something they’re frightened or unaware of*.” (44+-year-old non-Emirati) 003: “*And if you educate the young, hopefully they’ll kind of say oh call mum, you know, or grandma, I just had this done, and it’s making it more open and out there. And hopefully all the people will kind of get on board as well that way. Just kind of word of mouth*.” (30–44-year-old non-Emirati)	010: “*According to family history, and according to if they suspected something they will do [mammogram] yearly. And if normal, the first person, every two years they will do it*.” … 008: “*If they discover there is mass or something or some abnormal, any age*.” (30–44-year-old Emirati) 005: “*Below 40 would be recommended for ultrasound breast and mammogram above 40 is recommended for above 40, or if you have a history in your family … it can be before 40 also, will be advised by your doctor as for the history*.” (30–44-year-old non-Emirati) Researcher: “*Any plan for doing mammography*?” 006: “*Not for now, maybe later*.” … 007: “*If any symptom only we can do it. Otherwise, why? It’s not mandatory. If it is anything, meaning, if it is any suspicious means we can.*” (25–34-year-old non-Emirati)

**Table 3:** Breast cancer sources of information and advertisements.

Written/Printed Materials (breast cancer information)	Pamphlets, newspapers, magazines, posters, street boards, studies
Places (breast cancer information and campaigns)	Hospitals, primary health care centres, clinics, gynaecology clinics, schools and universities (for students and women), companies and workplaces (for staff), malls
People (breast cancer information and campaigns)	Doctors, gynaecologists, family, friends, ladies’ circles, breast cancer survivors.
Media (breast cancer campaigns advertisements)	Internet, social media, Facebook, Instagram, YouTube, Snapchat Mass media, televisions, news channels, radios SMS, WhatsApp

**Table 4:** Breast cancer promotions and campaigns.

Sources	Campaigns	Q. What Are the Current Available Sources of Breast Cancer Information and Campaigns in Ras Al Khaimah?
Places/Promotors	Hospitals, clinics, gynaecology clinics, schools, universities, hotels Breast clinic at Saqr Hospital (public), breast clinic in gynaecology clinic in RAK Hospital (private), Pink Caravan	Responses: 005: “*And we’re happy that this country [is] really supporting October. Almost every hospital will cover, every news channel, every day we were getting this, like lot of promotion*.” … 001: “*So in the days this, they are doing like activities for the women and they are giving like health education. And even in Saqr Hospital, they are doing like free mammograms who’s not local so they can go and do free mammogram for them*.” … 005: “*In many of the hospitals, even the private hospital they did mammogram free*.” (30–44-year-old non-Emirati women) 008: “*There is a group of medical team. They will come to the public health centre. They do screening for women*.” … 001: “*When I was in high school they make a meeting for this [breast cancer]. Also hospital for who want to go*.” (25–34-year-old Emirati woman)
Activities	Campaigns, programs, lectures, advertisements for breast cancer awareness, school health programs with visiting doctors (for children, women, or parents), advertisements for screening for males, broadcasted interviews with breast cancer survivors, breast cancer support groups	008: “*Now, thank God, the state does not fall short. There are awareness campaigns everywhere. There are awareness campaigns in hospitals, even in Red Crescent. Like, they do awareness, and they spread it on WhatsApp that there is so-and-so’s awareness campaign, or in areas they’ll send out that there are so-and-so’s awareness campaigns. They’ll go and do. You see, the state does not fall short.*” (44+-year-old Emirati)
Offers	Free breast self-examinations, free mammograms, vouchers for free screening access, screening in health camps, screening packages, screening at reduced prices, reduced screening costs if no health insurance, companies paying for staff screening	004: “*I’ve read in the newspaper that some clinics, some doctors, they offer free services for two three days in the labour* [construction] *camps. Or for the cleaners, women cleaners, and so on, who for sure cannot afford to do anything like this. And they go to those areas and they offer screenings for free*.” (44+-year-old non-Emirati woman) 007: “*A health medical camp. In that one mammogram and everything it’s free like that for who was there*.” (25–34-year-old non-Emirati woman)

**Table 5:** Women’s preferred media or activities for breast cancer information and campaigns.

Media	Preferred Media	Q. What are the Best Media to Receive Information Regarding Breast Cancer and Encourage Screening?
Media and Materials	Social media, SMS, WhatsApp, Pamphlets TV advertisements or street posters (not consensus)	Responses: 002: “*different ages you would find as well. Certain age will go for a pamphlet, and another age will go for the screen television with the medical information*” … 001: “*If I got something through the post like that, I might pick it up. Or not only that, I might just think, and it sounds awful, or more joking, and put it in the bin. Whereas if it came on my phone I would actually read that.*” 002: “*Well to get rid of it you’ve got to read it on your phone*.” (44+-year-old non-Emirati woman)
People/Places	Ladies groups, friends, families, people with experience (e.g., mothers, had breast cancer), breast cancer survivor stories, artists or influencers on social media, doctors, specialists Hospitals, primary health care centres	006: “*Opinion makers on the social media, especially for the youngest people … through the snapchat, Instagram, or Facebook. Because nowadays everybody is watching what opinion makers are doing, and if you would like to make awareness of something, you cannot miss this point because it’s 2019 point. And before we could make TV, radio, and pamphlet. Nowadays it won’t work*.” (30–44-year-old non-Emirati woman)
Promotions or Activities	Videos in clinics and waiting areas, encourage families and men to encourage women, home visits, forums, courses, lectures, school activities or subjects on women’s health Send reminders for appointments, follow-up examinations, or events	004: “*There are a lot of waiting areas here, and we tend to go alone to the doctors but within the local population, no. The ladies, or even the men they don’t go alone. And when the actual examination or treatment is done, yes they might be 5 or 10 min alone with the doctor, but there’s a lot of waiting, and then the relatives are waiting, so if there are pamphlets around or anything on TV where you run programs, informational programs, they could pick up on that*.” (44+-year-old non-Emirati woman)
Changes to Campaigns	More campaigns, awareness programs, and advertisements year round Free services and campaigns, increased access, longer campaigns, campaigns in rural regions, more centres with breast screening services and mammograms, group screenings	009: “*I did it in a group. They were younger than me. And it was normal and we encouraged each other. So we would enter, and each one had her turn. So it was fine, and we would exit smiling*.” 008: “*The outcome is sweet. It makes a person feel good*” 003: “*The idea is good, like, if it’s implemented. A group of women with all their appointments together is a beautiful idea*.” (44+-year-old Emirati women)
